# Motivations, dating app relationships, unintended consequences and change in sexual behaviour in dating app users at an Australian music festival

**DOI:** 10.1186/s12954-021-00493-5

**Published:** 2021-05-04

**Authors:** Shirali Garga, Meryl T. Thomas, Ashneet Bhatia, Aidan Sullivan, Franklin John-Leader, Sabrina W. Pit

**Affiliations:** 1 School of Medicine, University Centre for Rural Health, Western Sydney University, 61 Uralba Street, PO Box 3074, Lismore, NSW 2480 Australia; 2 Harm Reduction and Health Promotion Programs, HIV and Related Programs (HARP), North Coast Public Health, Mid-North Coast Local Health District, Lismore, Australia; 3 Faculty of Medicine and Health, University of Sydney, Sydney, Australia; 4 NSW Rural Doctors Network, Newcastle, Australia

## Abstract

**Background:**

Despite the popularity of dating apps, there remain scarce data on the motivations, consequences and their influence on sexual behaviour change in the Australian population.

**Objective:**

To explore motivations, dating app relationships, unintended consequences and change in sexual behaviour in dating app users at an Australian music festival.

**Methods:**

A cross-sectional study design was used. Festival patrons aged 18–30 at a major Australian music festival completed a paper-based survey. Logistic regression was used to identify which factors were associated with an increase in sexual partners since using dating apps.

**Results:**

The primary reasons for dating app use (*N* = 437) were boredom (59.7%), casual sex (45.1%) and casual dating (42.8%). A third of users used them at music festivals (33.8%, *n* = 432). A third of participants had used dating apps for more than 2 years (33.3%) and a third (33.0%) of users claimed to have changed their sexual behaviour after app use, including increased frequency of sexual activity (70.0%), number of sexual partners (57.1%) and sexual experimentation (42.1%). Dating app users tended not to discuss sexually transmitted infections (STI) status with a sexual partner regardless of whether they had met them on an app or not: 38.5% would ‘never’ and 36.9% would ‘sometimes’ have safe sex discussions with partners met via apps. Condoms were ‘always’ used for 36.9% of dating app users when meeting partners via dating apps, compared to 29.9% met by other means. 8.6% of dating app users reported having contracted STIs, and 2.8% had unwanted pregnancies with those met on dating apps. After adjusting for socio-demographics, those who had an STI after engaging in sexual activity with a person met via a dating app had 2.4 times the odds of reporting an increase in sexual partners, and those who had used a dating app for over 2 years had twice the odds of reporting an increase in sexual partners. When condom use was entered into the model, those that ‘often’ or ‘sometimes’ used a condom with a new dating app partner were twice as likely to report an increase in sexual partners since using dating apps, compared to those who ‘always’ used a condom with a new dating app partner. Sexual orientation and STI discussions with a new sexual dating app partner were not associated with an increase in dating app partners.

**Conclusion:**

Dating app usage is common and users report increased sexual activity, sexual partners and experimentation. STI discussions with potential partners and condom use remained low regardless of how partners were met and despite an increase in sexual partners since using dating apps. Given the high-risk nature of individuals that utilise dating apps, safe sex discussion, including STIs, pregnancies and condom use should be promoted to improve sexual health outcomes.

## Background

Online dating has existed for over 20 years now. Initially, dating websites were accessed via computers; however, since smartphones have become ubiquitous in society, increasing numbers of dating applications have become available [1]. Many of these applications utilise global positioning system technology to connect users by physical proximity, known as geosocial networking dating apps [2]. Users can filter matches for their desired demographic characteristics including age and location, with some applications such as Hinge offering many more filters including ethnicity; religion; family plans; height; politics and smoking, marijuana and drug-taking status [3].

### Reasons for using dating apps

Young people have different reasons for using dating apps but this varies per context. The predominant motivators of dating app use in a 2018 survey of 409 American university students were found to be for fun and to meet people [4]. Other studies found the main motivator to be forming romantic and sexual connections [5, 6]. Though not found to be a major motivator, Orosz et al. [7], Sumter et al. [8] and Ranzini and Lutz [9] included self-validation as a reason for dating app use in their studies. This may be linked to low self-esteem and is an important correlate that warrants further investigation in an Australian population especially in the context of a 2020 Australian study that showed dating app users report higher levels of depression, anxiety and stress [10].

### Relationships, sexual health behaviours and risks

To date, many studies of meeting partners online have recruited men who have sex with men (MSM) and used sampling strategies that are unlikely to be representative of a predominantly heterosexual population, such as targeting attendees of gay venues, or sexual health care clinics [11, 12]. However, the use of dating apps extends far beyond the MSM population. Indeed, a recently published study looking at 2012–2013 data found that more than a third of Australian adults (*n* > 20,000) had searched for potential partners online [1]. A Brazilian study found that women are far less likely than men to have a condom with them, when engaging in casual sex, leaving them in the vulnerable position of relying on their sexual partner to supply adequate protection [13]. Thus, both heterosexual and non-heterosexual dating app users warrant targeted health promotion.

Several international studies have explored whether dating apps promote sexually promiscuous behaviour and the results are mixed. These mixed results can be explained by many factors including cultural differences and sexual orientation. Two US-based studies found that among MSM, dating app users reported more sexual contacts and casual sex partners than non-users [5, 6]. Choi et al. found that among students at four Hong Kong universities, the use of dating apps for over a year was associated with illicit drug use during sexual intercourse [14]. Knox et al. found that Chinese MSM who used dating apps were 2.5 times more likely to have unprotected anal sex with a partner met offline than online [15]. An analysis by Albury et al. of the depiction of dating apps in Australian and overseas media found a high prevalence of articles framing dating apps as dangerous to physical, mental and sexual health. Many of these articles were based upon expert opinion and individual user experiences rather than validated research. This included articles drawing associations between dating app use and increased susceptibility to STIs [16]. Dating apps very well may pose health risks to young Australians; however, the level of risks and associated factors needs to be further explored. Our study aims to add to the literature by providing objective findings on sexual partners met via dating apps, increase in sexual behaviour due to app use and factors associated with increased sexual activity since using apps. Indeed, Albury et al. [16] concluded that more work is needed to examine the emerging dating and hook-up app data cultures from the perspectives of users themselves. They suggest that methods beyond standard qualitative interview or focus group approaches are needed. Our study assists in addressing this gap by using quantitative survey data to explore and better understand dating app users and opportunities for improving sexual health.

### Music festivals and risky health behaviour

Furthermore, music festival patrons are known to be at high-risk from a public health perspective including excessive drinking, drug use, sexting and risky sexual behaviour. Firstly, Jenkinson et al. who conducted a study at an Australian music festival found that 89% of their participants identified as heterosexual and 84% were sexually active [17]. They found that among festival attendees aged between 16 and 29, 27% were at high risk of STIs due to unprotected sex with new or casual partners in the last 12 months [17]. Secondly, Hall et al. found that 94% of young festival patrons had been under the influence of drugs or alcohol during sex in the last 12 months [18]. Drug and alcohol use has been linked to unsafe sexual behaviour and condom use problems [18]. Given that alcohol and drug use is very common at festivals, this is a cause of concern. A 2018 study found that 73.4% of festival patrons reported that they had used illicit drugs in the past 12 months [19]. In Norway, 10% of mainstream festival goers reported illegal substance use within the past 30 days, five times higher than the rate of cannabis use among the general Norwegian population [20]. Another 2018 study found that among festival attendees, the median number of alcoholic drinks consumed in the last 24 h was 12 standard drinks, which is twice the number of standard drinks viewed as binge drinking [21]. Thirdly, dating app usage can also be linked to sexting. Whilst it is acknowledged that sexting can have a positive impact, a 2019 study identified that the more unsolicited sexts were received, the higher the distress levels were among the festival respondents and that sexting can be perceived as risky [22]. Indeed, a US-based study among college students also found that 80% perceived sexting as risky [23]. Sexting is common among festival patrons with a study finding that 53.1% of respondents had sent a sexually explicit message, 43.1% had sent a sexually explicit image, 61.2% had received a sexually explicit message, and 55.1% had received a sexually explicit image [22]. In summary, given the high-risk environments, music festivals form an ideal place to further explore dating apps to improve positive sexual health outcomes.

We acknowledge that targeting a venue with high levels of risky behaviours at an Australian music festival is potentially not completely representative of the heterosexual population. However, we have done repeated studies in the same music festival scene annually and have consistently identified high levels of heterosexual sexual orientation among respondents: 88.9% in 2015 [18], 90.4% [19] in 2016 and 89.4% in 2018 [22]. Thus, music festivals provide a venue where sexual behaviours can be investigated in a population irrespective of sexual orientation. In summary, given the high-risk profile of music attendees, more in-depth understanding of the reasons for using dating apps, dating app relationships and the impact of dating apps on sexual health behaviour among festival attendees is warranted from a public health perspective. To our knowledge, this has not been investigated before in a high-risk young adult population at a music festival.

### Dating apps and positive sexual health promotion at music festivals

Understanding the behaviours and characteristics of dating-app users at festivals can support development of positive sexual health promotion activities. Tavares et al. found that 29.2% of male dating app users had received some form of safe sex information through the apps compared to only 3% of women [13]. A possible explanation is that women may simply not notice the messages or are not targeted. A review of 60 dating apps found that only 9 dating apps had sexual health content and seven of these only targeted MSM [2]. Our study can potentially be used to reduce harm for young people using dating apps through health promotion interventions, such as safe sex campaigns both on dating apps and at music festivals themselves to improve sexual health outcomes. With geocoded locations, dating apps now also have the opportunity to promote safe sex at specific festivals locations.

Therefore, this study aims to explore motivations, dating app relationships, unintended consequences and factors associated with change in sexual behaviour in dating app users at an Australian music festival. Given the high-risk population, we hypothesise that dating app users who report an increase in sexual partners are less likely to report condom use with new sexual partners met via a dating app and less likely to discuss STIs with new dating app partners.

## Methods

### Study design

A cross-sectional survey using convenience sampling was conducted in 2019. The only inclusion criteria were that participants must be between 18 and 30 years old and not visibly intoxicated. No incentives were provided.

### Data collection

Data collection took place at a large three-day music festival in New South Wales, Australia. Festival goers who visited a permanent sexual health promotion stall within the campgrounds were invited to participate, and people who were perceived to be between 18 and 30 years of age were invited to take part. The festival is mainly attended by young people. We had ethics approval to invite people aged between 18 years and over. Thirty years was set as the upper boundary to have a relatively homogenous study population and has been consistently used in previous years at the same venue by the authors. Participants were provided with a participant information sheet and were able to ask questions prior to participation to make an informed decision on participation. If people agreed to take part, they were invited to complete the survey. Prior to survey completion, participants were asked to read participate information sheet and survey completion was taken as consent. Participant anonymity was maintained as completed surveys were placed into closed boxes and did not ask for any identifying information. The number of patrons who refused to take part was not documented.

The survey was developed in consultation with sexual health and public health experts and was pilot-tested with thirteen university students who reflect the target population. The survey was further defined and approved by the Western Sydney University Human Research Ethics Committee (H11327). All methods were carried out in accordance with relevant guidelines and regulations.

### Outcome measures

Participants were asked their reasons for using dating apps (‘To find a long-term relationship’, ‘Casual dating’, ‘Casual sex’, ‘To make friends’, ‘For a self-confidence boost’, ‘Out of boredom or just for fun, no intentions of meeting potential matches’, ‘Other’). The categories for reasons for app use were taken from Orosz et al. [7] who investigated ‘sex, love, self-esteem enhancement and boredom’ as the four motivators for Tinder use. Our questionnaire added ‘making friends’ as a potential motivator as this was included in several other studies [5, 9]. Participants were also asked which dating apps were used (‘Tinder’, ‘Coffee meets bagel’, ‘Grindr’, ‘Bumble’, ‘OkCupid’, ‘Hinge’, ‘Plenty of fish’, ‘Happn’, ‘RSVP’, ‘eHarmony’, ‘Zoosk’, ‘Other’), amount of time using dating apps (‘Less than a month ago’, ‘1–6 months ago’, ‘6 months–1 year ago’, ‘1–2 years ago’, ‘2–4 years ago’, ‘4 + years ago’, how many people they’ve met with face-to-face from an app, number of serious relationships with people met via apps and the proportion of sexual partners met via apps (‘All of them’, ‘More than half’, ‘Less than half’, None’). They were also asked about any change in sexual behaviours following app use (‘yes’, ‘no’). If they said yes, they were asked what kind of change (‘Increased frequency of sexual activity’, ‘Increased number of sexual partners’, ‘Increased sexual experimentation’, ‘Other’). They were also asked to compare condom use and discussion about STI status with partners met from an app or not via an app (‘Always’, ‘Often’, ‘Sometimes’, ‘Never’, ‘Not applicable’) and whether they had contracted an STI or had an unwanted pregnancy with a sexual partner met via an app.

## Data analysis

SAS 9.3 (SAS Institute, Cary, NC, USA) was used to analyse the data. Simple descriptive statistics are provided. Logistic regression was used to calculate crude ratios to determine the associations between self-reported increase of sexual partners since starting using dating apps with the following dependent variables: age, gender, sexual orientation, relationship status, length of time of using dating apps, having an STI due to sexual activity with a new partner met via a dating app, STI discussions with a new sexual dating app partner and condom use with a new sexual partner met via dating app. The significance level was set at 0.05. Three multivariate models were analysed based on variables that were statistically significant in the bi-variate analyses. Model 1 included only socio-demographics; model 2 included model 1 plus dating app length and contracting an STI with a dating app partner. Model 3 included models 1 and 2, plus frequency of condom use with new sexual partners met via dating apps. Adjusted odds ratios and their 95% confidence intervals are presented.

## Results

### Demographics

As shown in Table 1, the majority of dating app users were 21–24 years old (48.0%, 215/418), closely followed by 18–20-year-olds (42.9%). There was a slight skew towards female participants, with 65.1% of dating app users being female and 86.6% identifying as heterosexual, whilst 11.3% identified as either homosexual or bisexual.

**Table 1:** Demographics of dating app users

Characteristics	Frequency (%)
Dating app use (*N* = 862)	448 (52.0%)
*Age (N* = *448)*	
18–20	192 (42.9%)
21–24	215 (48.0%)
25–30	41 (9.2%)
*Gender (N* = *447)*	
Female	291 (65.1%)
Male	156 (34.9%)
*Sexual orientation (N* = *448)*	
Heterosexual	388 (86.6%)
Homosexual	19 (4.2%)
Bisexual	32 (7.1%)
Other	9 (2.0%)

### Motivations, dating app relationships and usage characteristics

A slight majority of music festival attendees (52.0%, *N* = 862) were dating app users (Table 2). The primary reason for dating app use (*N* = 437) was boredom (59.7%), casual sex (45.1%) and to casually date (42.8%). The primary reasons in participants who did not use dating apps (*N* = 405) were due to existing relationships (61.5%), and a preference to meet people through other means (35.6%). Of the participants who used dating apps, a third used them at music festivals (33.8%, *n* = 432), and the major motivators for using them at festivals were for casual sex (55.9%), boredom (51.7%) and to make friends (41.4%).

**Table 2:** Motivations, dating app relationships and usage characteristics

Characteristics	Frequency (%)
Dating app use (*N* = 862)	448 (52.0%)
Dating app use at festivals (*N* = 432)	146 (33.8%)
*Reasons for not using dating apps (N* = *405)*	
In a relationship	249 (61.5%)
Prefer to meet people off apps	148 (36.5%)
Not dating	14 (3.5%)
Don’t like dating apps	13 (3.2%)

Reasons for using dating apps	Reasons in general (*N* = 437)	At music festivals (*N* = 145)
Boredom	261 (59.7%)	75 (51.7%)
Casual sex	197 (45.1%)	81 (55.9%)
Casual dating	187 (42.8%)	21 (14.5%)
Self-confidence boost	146 (33.4%)	33 (22.8%)
To find a long-term relationship	94 (21.5%)	11 (7.6%)
To make friends	71 (16.2%)	60 (41.4%)
Other	6 (1.4%)	4 (2.8%)
*Dating apps used (N* = *444)*		
Tinder	431 (97.1%)	
Bumble	181 (40.1%)	
Hinge	38 (8.6%)	
Grindr	14 (3.2%)	
Plenty of fish	12 (2.7%)	
OkCupid	5 (1.1%)	
Happn	8(1.8%)	
Coffee meets bagel	2 (0.5%)	
Eharmony	4 (0.9%)	
RSVP	0 (0.0%)	
Zoosk	0 (0.0)	
*Period of dating app usage (N* = *438)*		
Under 6 months	90 (20.5%)	
6–12 months	76 (17.4%)	
1–2 years	126 (28.8%)	
More than 2 years	146 (33.3%)	
*Sexual partners met *via* dating app (N* = *442)*		
None	226 (51.1%)	
Less than half	55 (12.4%)	
More than half	128 (29.0%)	
All	33 (7.5%)	
People met face-to-face from dating app (*N* = 444) [Mean, Median (Min, Max)]	4.52, 2 (0, 50)	
Number of long-term relationships (*N* = 444) [Mean, Median (Min, Max)]	0.37, 0 (0, 12)	
Specified radius (km) on dating apps (*N* = 444) [Mean, Median (Min, Max)]	41.91, 30 (1, 1000)	

Tinder was the most used app (97.1) followed by Bumble (40.1%). A third of participants had used dating apps for more than 2 years (33.3%), followed by 1–2 years (28.8%). The median number of people met face-to-face from dating apps was 2, but the median number of long-term relationships was 0. Whilst 51.1% of the participants had met no sexual partners via dating apps, 29.0% had met more than half of their sexual partners via dating apps.

### Change in sexual behaviour, safe sex and unintended consequences among dating app users

Table 3 describes the behaviours of dating app users. Remarkably, a third (33%) of users claimed to have changed their sexual behaviour after app use. The major changes being increased frequency of sexual activity (70%) and increased number of sexual partners (57.1%). Dating app users tended not to discuss STI status with a sexual partner regardless of whether they had met them on an app or not. A significant proportion (38.5%) of users stated they had ‘never’ had safe sex discussions with partners met via apps, but 36.9% would ‘sometimes’ have these discussions with partners not met via dating apps. Condoms were ‘always’ used for 36.9% of dating app users when meeting partners via dating apps, compared to 29.9% for partners not met via dating apps. Of the participants, 8.6% reported that they had contracted STIs and 2.8% had unwanted pregnancies with those met on dating apps.

**Table 3:** Change in sexual behaviour, safe sex and unintended consequences among dating app users

Characteristics	Frequency (%)
Change in sexual behaviour after app use (*N* = 442)	146 (33.0%)
*Specific behaviour changes (N* = *140)*	
Increased frequency of sexual activity	98 (70.0%)
Increased number of sexual partners	80 (57.1%)
Increased experimentation	59 (42.1%)
Other	2 (1.4%)

Discussion of STI status with a sexual partner	Met via app (*N* = 325)	Not met via app (*N* = 385)
Always	52 (16.0%)	63 (16.4%)
Often	41 (12.6%)	56 (14.5%)
Sometimes	107 (32.9%)	142 (36.9%)
Never	125 (38.5%)	124 (32.2%)

Condom use with a sexual partner	Met via App (*N* = 325)	Not met via app (*N* = 391)
Always	120 (36.9%)	117 (29.9%)
Often	72 (22.2%)	94 (24.0%)
Sometimes	92 (28.3%)	117 (29.9%)
Never	43 (13.2%)	63 (16.1%)
Unwanted pregnancy from dating app partner (*N* = 429)	12 (2.8%)	
STI contracted from dating app partner (*N* = 429)	37 (8.6%)	

### Factors associated with dating app users reporting an increased number of sexual partners since using dating apps

Crude odds ratios (Table 4) showed that the following variables were associated with a self-reported increase of sexual health partners since starting using dating apps: older age, being male, casual dating, using dating apps for over 2 years, having an STI after sexual activity with a dating app partner and using a condom with a new sexual dating app partner ‘often’ or ‘sometimes’. Sexual orientation and STI discussions with a new sexual dating app partner were not associated with an increase in dating app partners.

**Table 4:** Crude and adjusted odds ratios of factors associated with dating app users reporting an increased number of sexual partners since using dating apps (*N* = 448)

Dating app users reporting an increased number of sexual partners since using dating apps	Yes (*N* = 80)	No (*N* = 356)	Crude odds ratios			Model 1—socio-demographics			Model 2—socio-demographics + dating app length + STI			Model 2—socio-demographics + dating app length + STI + condom use		
	*n* (%)	*n* (%)	Odds ratio	95% CI	*P* value	Odds ratio	95% CI	*P* value	Odds ratio	95% CI	*P* value	Odds ratio	95% CI	*P* value
*Age*														
18–20	20 (10.8)	166 (89.2)	1	–	–	1	–	–	1	–	–	1	–	–
21–24	46 (22.0)	163 (78.0)	2.3	1.3–4.1	**0.0033**	2.1	1.2–3.8	**0.0110**	1.7	0.93–3.3	0.0813	1.6	0.8–3.2	0.1430
25–30	14 (34.2)	27 (65.8)	4.3	1.9–9.5	**0.0003**	4.0	1.7–9.1	**0.0009**	3.3	1.4–7.9	**0.0062**	2.5	0.96–6.5	0.0602
*Gender*														
Male	44 (15.5)	240 (84.5)	1.7	1.0–2.8	0.0364	1.8	1.1–3.1	**0.0263**	1.6	0.95–2.8	0.0767	1.5	0.8–2.6	0.1985
Female	36 (23.7)	116 (76.3)	1	–	–	1	–	–	1	–	–	1	–	–
*Heterosexual*														
Yes	64 (17.0)	313 (83.0)	1	–	–	–	–	–	–	–	–	–	–	
No	16 (27.1)	43 (72.9)	1.8	0.96–3.4	0.064	–	–	–	–	–	–	–	–	–
*Relationship status*														
Single and not dating	24 (12.8)	164 (87.2)	1	–	–	1	–	–	1	–	–	1	–	–
Casual dating	39 (27.9)	101 (721.1)	2.6	1.5–4.6	**0.0008**	2.9	1.6–5.2	**0.0005**	2.4	1.3–4.4	**0.0059**	2.3	1.2–4.5	**0.0127**
Exclusive	17 (16.2)	88 (83.8)	1.3	0.7–2.6	0.4188	1.2	0.6–2.4	0.6029	1.0	0.51–2.2	0.9008	1.3	0.6–2.09	0.497
*Period of dating app usage*														
Less than 6 months	17 (19.1)	72 (80.9)	1	–	**–**	–	–	–	1	–	–	1	–	–
6–12 months	6 (8.0)	69 (92.0)												
1–2 years	14 (11.4)	109 (88.6)												
More than 2 years	43 (29.9)	101 (70.1)	2.9	1.8–4.7	** < 0.0001**	–	–	–	2.0	1.2–3.5	**0.0121**	2.1	1.2–3.8	**0.0136**
*Had STI after engaging in sexual activity with a person met *via* a dating app*														
Yes	13 (35.1)	24 (64.9)	2.7	1.3–5.5	**< 0.0001**	–	–	–	2.4	1.1–5.1	**0.0287**	1.9	0.86–4.3	0.1145
No	65 (16.9)	319 (83.1)	1	–	–	–	–	–	1	–	–	1	–	–
*STI discussion with new sexual partner met *via* dating app*														
Always	11 (21.2)	41 (78.8)	1.2	0.5–2.6	0.7264	–	–	–	–	–	–	–	–	–
Often	12 (30.0)	28 (70.0)	1.8	0.8–4.2	0.1405	–	–	–	–	–	–	–	–	–
Sometimes	29 (27.6)	76 (72.4)	1.6	0.9–3.1	0.1189	–	–	–	–	–	–	–	–	–
Never	23 (18.9)	99 (81.1)	1	–	–	–	–	–	–	–	–	–	–	–
*Condom use with new sexual partner met *via* dating app*														
Always	20 (16.8)	99 (83.2)	1	–	–	–	–	–	–	–	–	1	1	1
Often	24 (34.8)	45 (65.2)	2.6	1.3–5.3	**0.0058**	–	–	–	–	–	**–**	1.9	1.0–3.7	**0.041**
Sometimes	24 (27.6)	63 (72.4)	1.9	0.96–3.7	0.0644									
Never	6 (14.0)	37 (86.0)	0.8	0.3–2.2	0.6627	–	–	–	–	–	–	0.64	0.21–1.9	0.4254

Bold means statistically significant

Model 1 shows that people aged 21–24 and 25–30 years were, respectively, twice (OR 2.1, 95% CI 1.2–3.8) and four times (OR 4.0, 95% CI 1.7–9.1) as likely to report an increase in sexual partners due to dating apps compared to people aged 18–20. Men were twice (OR 1.8, 95% CI 1.1–3.1) as likely to report an increase in sexual dating app partners, and compared to people who were single those that were casually dating were three times (OR 2.9, 95% CI 1.6–5.2) as likely to report an increase in sexual dating app partners.

Model 2 shows that being aged between 25 and 30 years and casual dating remained statistically significant when the model was adjusted for the length of time a dating app has been used and STI contraction after having sex with a dating app partner. The period of dating app usage was collapsed into two categories to reduce the number of variables in the model given the low numbers in the outcome measure, and showed that people who had used a dating app for 2 years or more had twice the odds of having an increase in sexual partners than those who had used it for less than 2 years (OR 2.0, 95% CI 1.2–3.5). Similarly, people who had an STI after engaging in sexual activity with a person met via a dating app had 2.4 times the odds of reporting an increase in sexual partners (OR 2.4, 95% CI 1.1–5.1).

Model 3, the full model, included models 1 and 2 and self-reported frequency of condom use with a new sexual partner met via dating apps and demonstrates that age, gender and contracting and STI from a dating app partner were no longer statistically significant. Furthermore, the final model showed that casual daters had twice the odds (OR 2.3, 95% CI 1.2–4.5) of having an increase of sexual partners compared to single people, and those who had used a dating app for over 2 years also had twice the odds of reporting an increase in sexual partners (OR 2.1, 95% CI 1.2–3.8). Compared to those who always use a condom with a new dating app partner, those that ‘often’ or ‘sometimes’ used a condom with a new dating app partner were twice as likely to report an increase in sexual partners since using dating apps.

## Discussion

There were several significant findings elicited from this study. The first being that one-third (33%) of users noticed a change in their sexual behaviour after app use. The major changes being an increase in frequency of sexual activity (70%), number of sexual partners (57.1%) and experimentation (42.1%). Despite increased sexual behaviours, app users tended not to discuss STIs with a sexual partner regardless of whether they had met them on an app or not, and condoms were ‘always’ used by only 36.9% of dating-app users when meeting partners via dating apps. Primary reasons for using dating apps at festivals were boredom, casual sex and making friends.

Multivariate analyses showed that after adjusting for age, gender and relationship status that people who had an STI after engaging in sexual activity with a person met via a dating app had 2.4 times the odds of reporting an increase in sexual partners. Similarly, those who had used a dating app for over 2 years had twice the odds of reporting an increase in sexual partners. However, STI contraction was no longer associated with an increase in sexual partners since using dating apps after condom use was entered into the model. Compared to those who ‘always’ use a condom with a new dating app partner, those that ‘often’ or ‘sometimes’ used a condom with a new dating app partner were twice as likely to report an increase in sexual partners since using dating apps. The low rates of using condoms with new sexual dating app partners have sexual health implications among this group of young dating app users, as notably 8.6% reported contraction of STIs and 2.8% reported unwanted pregnancies. The rate of STI infection among dating app users was comparable to a recent Australian study with the same age range (7.4%) [24]. Harm reduction practices could assist in promoting safe sex among a group that has increased their sexual activity due to using dating apps. Specifically, long-term dating app users could be targeted for health promotion activities using condoms, STI discussion and unintended pregnancies. Choi et al. [25] also found that app users who used apps for more than 12 months were likely to have more lifetime sexual partners as well as more sexual partners in the last three months compared to people who had used apps for less than 12 months among university students.

Our study also described a significant lack of STI discussion among dating app users regardless of whether they had met via dating apps. Only 16.0% of respondents always discussed STI status with a sexual partner met through a dating app, an almost identical rate of 16.4% was recorded for discussion with partners met via other means. Importantly, the multivariate analyses showed that an increase in sexual partners since using dating apps was not associated with STI discussions with new sexual partner met via dating apps. This indicates there is room for an increase in health promotion initiatives encouraging people to discuss their STI status prior to sexual activity. Dating apps can contribute to these health promotion activities to reduce further harm among this at-risk group. The large majority of dating app users support the use of safe sex messages (88%) [26], suggesting that this is an easy way to target an at-risk group.

Our study found that 2.8% of respondents had an unintentional pregnancy due to sexual intercourse with someone met via a dating app. However, a 2016 Australian study reported that participants between the ages of 18–23 had a much higher rate of 13.5% rate of unplanned pregnancy [27]. The difference can be explained by the fact that the latter study only included women, and the survey was advertised as a study about unintended pregnancies, and thus was probably biased towards recruiting women who had unintended pregnancies. A third of the women in the study [27] with unintended pregnancies were using the withdrawal method. Therefore, safe sex messages on dating apps should also warn about how to avoid unwanted pregnancies. Furthermore, timing of safe sex dating app messages could centre around known large festival periods and be location based as stated previously many applications use global positioning system technology to connect users by physical proximity.

To our knowledge, this is the first study that has investigated the reasons for using dating apps specifically at festivals and outside of festivals. A third of participants (33.8%) used dating apps at music festivals. The motivators for dating-app use at festivals varied as compared to life outside of music festivals. The primary difference being a significantly higher proportion electing dating app use at festivals to make friends (41.4% compared to 16.2%) and a lower proportion to casually date (14.5% compared to 42.8%) yet a higher proportion using the app for casual sex (45.1% vs. 55.9%). There are limited existing data on the motivations of dating app use at music festivals and the causal link of this to risky sexual behaviours. This is reflective of other literature regarding dating app use such as Goedal et al. [5] and Rice et al. [6], despite these studies focusing on MSM populations. This may suggest that motivations between populations may be similar.

The data also showed that 49% of dating app users had met sexual partners via dating apps, with 35% having met more than half of their sexual partners via dating apps. This further highlights that dating apps are an ideal platform to promote positive sexual health among those who have multiple sexual partners overtime when using dating apps.

## Limitations and strengths

The study has several limitations, the prevalence of selection bias due to convenience sampling, the inherent weaknesses in self-reported data and also the lack of cause and effect shown due to the study’s cross-sectional design.

There is also a clear skew towards female participants in this cohort as compared to what is true of music festivals and the general Australian populace which may affect the generalisability of the results to other groups. Participants may have been under the influence of drugs and alcohol despite an attempt to exclude these participants by testing early in the day and not approaching those who were discernibly intoxicated.

A major strength of the study lies in the huge scale of the music festival from which the sample was selected. Given that the numbers in attendance were so massive, the recruited participants were more likely to be representative of the wider Australian populace. Our previous research identified that festival goers who identify as LBGTIQ + and those not in an exclusive relationship are more likely to use dating apps [26]. However, 89.3% of participants in the study identified themselves as heterosexual. This is reflective of Australian youth, a survey of 1,168 Australian students found that 89% were heterosexual [28]. Dating app use is also most prevalent in the 18–24-year-old age group [29] and 90.2% of our survey respondents were in the 18–24-year-old age bracket.

## Conclusion

Dating app usage is common and users tend to report increased sexual activity and number of sexual partners and experimentation. The primary motivators for dating app use are boredom, casual sex and casual dating. However, at music festivals, dating app patrons are more interested in making friends than finding potential dating partners. Discussions of STIs with potential partners and condom use remained low. Given the high-risk nature of individuals that utilise dating apps, safe sex discussion and condom use should be promoted to improve sexual health outcomes.

## Data Availability

The datasets are not available from the corresponding author due to the consent being provided for participation in the specific study only.

## Abbreviation

STI: Sexually transmitted infections
